# *IGF2-H19* Locus Expression Profile and Biomarker Potential in Oral Cancer

**DOI:** 10.3390/biology15151221

**Published:** 2026-07-23

**Authors:** Goran Stojkovic, Milica Jaksic Karisik, Nikola Todorovic, Marija Savic Veselinovic, Jovana Rosic Stojkovic, Katarina Zeljic

**Affiliations:** 1 Faculty of Medicine, University of Belgrade, 11000 Belgrade, Serbia; drgoranstojkovic@gmail.com (G.S.); jovana.rosic@med.bg.ac.rs (J.R.S.); 2 Clinic for Otorhinolaryngology and Maxillofacial Surgery, University Clinical Center of Serbia, 11000 Belgrade, Serbia; nikola.todorovic89@hotmail.com; 3 School of Dental Medicine, University of Belgrade, 11000 Belgrade, Serbia; milica.jaksic@stomf.bg.ac.rs; 4 Faculty of Biology, University of Belgrade, 11000 Belgrade, Serbia; marijas@bio.bg.ac.rs

**Keywords:** *IGF2*, *H19*, hsa-miR-483, hsa-miR-675, oral cancer

## Abstract

*IGF2-H19* locus is important for normal development and growth, and its deregulation has been implicated in various cancers, with conflicting data in oral cancer. *IGF2* and *H19* expression was analyzed in oral cancer (SCC-25, SCC-15) and normal keratinocytes (HaCaT) cell lines, as well as in cancer tissue and adjacent non-cancerous tissues from 55 patients with oral cancer. The expression of *IGF2* and *H19* was increased in oral cancer cell lines compared with control cell line. In clinical samples, expression of the *IGF2-H19* locus and its associated hsa-miR-675-5p was significantly lower in cancer tissue compared with adjacent non-cancerous tissue. *IGF2*, *H19*, and hsa-miR-675-5p demonstrated moderate ability to discriminate between oral cancer and non-cancerous tissues, while the combined analysis of all three molecules improved diagnostic performance. Our results suggest that *IGF2-H19* and hsa-miR-675-5p may have potential as molecular biomarkers for discriminating between oral cancer and non-cancerous tissue, but do not appear to have prognostic value.

## Simple Summary

Oral cancer is one of the most common head and neck malignancies, and its early and sensitive detection remains a major clinical challenge. In this study, we investigated several molecules involved in the control of normal cell growth and development to determine whether they could help distinguish cancer tissue from healthy tissue and potentially help in determining prognosis of patients. We analyzed their expression in oral cancer cells grown in the laboratory and in tissue samples obtained from patients with oral cancer. We found that the levels of these molecules were significantly different between non-cancerous and cancer tissues. When all three molecules were analyzed together, they showed improved ability to identify oral cancer. However, these molecules were not associated with predicting disease outcome. Our findings suggest that these biological markers may be useful for improving the detection of oral cancer and could contribute to the development of more accurate diagnostic tools in the future. Further studies involving larger numbers of patients are needed to confirm these results.

## 1. Introduction

Head and neck squamous cell carcinoma (HNSC) is a heterogenous group of cancers arising in the head and neck region, including oral cavity cancer [1]. Oral cancer (oral squamous cell carcinoma, OSCC) is the most common subtype within this group, accounting for approximately 90% of all HNSC cases [2]. The global incidence of oral cancer is increasing [3], particularly in Europe and among younger populations [4]. Lifestyle factors such as smoking and alcohol consumption, as well as environmental factors like infection with high-risk human papillomavirus types, are considered important triggers for malignant transformation of the squamous cells in oral cavity [2]. Oral cancer is usually diagnosed at an advanced stage of the disease, which significantly worsens the prognosis [5]. Surgical treatment is the primary therapy often followed by radiotherapy with or without chemotherapy [6]. However, local recurrences occur in up to 30% of cases indicating the need for more sensitive and specific methods to distinguish oral cancer from normal oral mucosa. This indicates the importance of integrating molecular pathology approach alongside conventional histopathological analysis. Furthermore, the identification of prognostic biomarkers as well as discovery of novel therapeutic targets is essential to improve oral cancer patient outcomes and enable more personalized treatment in the future.

*IGF2-H19* locus (11p15.5) represents an imprinted locus encoding growth factor IGF2 and long noncoding RNA (lncRNA) H19, in which paternal *IGF2* and maternal *H19* expression are tightly regulated, while dysregulation of this locus has been implicated in the pathogenesis of multiple human cancers [7]. Proper tissue growth during embryogenesis is enabled due to balanced expression of *IGF2* and *H19*. Loss of imprinting leads to overexpression of *IGF2*, deregulation of *IGF2-H19* and consequently to malignancy. *IGF2* expression was increased in adrenocortical carcinoma [8], hepatoblastoma [9], and other cancer types, thus considered as protooncogene. The available evidence on *IGF2* in OSCC remains limited. Previous studies conducted on two commercial cell lines, SCC-4 and SCC-25, both derived from tongue epithelium, have reported increased *IGF2* expression. However, these studies did not investigate the biological significance of *IGF2* overexpression or clarify its potential role in OSCC development and progression [10]. *H19* is also mainly recognized as a protooncogene, but there are data suggesting its tumor suppressor role depending on the cellular context, indicating a dual role for *H19* [11]. There are conflicting data on *H19* expression in oral cancer [12,13]. A recent systematic review confirmed that *H19* is dysregulated in OSCC. However, the available studies report conflicting results, with some demonstrating increased and others decreased *H19* expression. Therefore, *H19* may not be suitable as a standalone biomarker in OSCC [14].

Micro RNAs (miRNAs) are encoded within *IGF2-H19* locus. MiRNAs are small non-coding RNA molecules, ~24nt in length, with an important role in the regulation of vital cellular processes, such as differentiation and apoptosis. miRNAs regulate expression of target genes by suppressing expression posttranscriptionally either by mRNA degradation or translational repression. It is known that hsa-miR-483 and hsa-miR-675 miRNAs are transcribed from the *IGF2-H19* locus. Hsa-miR-483-3p is located within the second intron of the *IGF2* gene, while hsa-miR-675-5p is embedded within the first exon of *H19* [7]. Aberrant expression of these miRNAs has been detected in different cancer types [8,15,16,17,18,19].

To the best of our knowledge, *IGF2-H19* locus and its mRNA, lncRNA and miRNAs have not been extensively studied in oral cancer. Considering the role of *IGF2-H19*, we hypothesized that molecules transcribed from the *IGF2-H19* locus contribute to oral cancer development cooperatively, and thus mRNA, lncRNA and miRNAs generated from the locus might be promising molecular candidates for sensitive discrimination of cancer from non-cancerous tissue as well as prognosis in oral cancer patients. This study aimed to comprehensively analyze expression levels of *IGF2* and *H19* and their associated miRNAs in oral cancer cell lines, publicly available datasets and oral cancer clinical samples and to evaluate their association with clinicopathological characteristics and biomarker potential. Thus, this study fills a gap in knowledge regarding *IGF2-H19* locus-related transcripts and their biomarker potential in oral cancer.

The main finding of the current study is that the combination of *IGF2*, *H19*, and hsa-miR-675-5p shows good performance in distinguishing between oral cancer and non-cancerous tissue.

## 2. Materials and Methods

### 2.1. Cell Cultures

In this study, three cell cultures were utilized, including the human oral squamous cell carcinoma (OSCC) cell lines SCC-25 and SCC-15 obtained from the American Type Culture Collection (ATCC CRL-1628™, ATCC CRL-1623™) as well as immortalized human keratinocytes HaCaT (CLS/Cytion, catalog number 300493, Eppelheim, Germany). HaCaT cells represented a healthy keratinocyte cell line and were used as the control group in this study.

Cells were cultured in T25 cell culture flasks using a complete medium consisting of Dulbecco’s Modified Eagle Medium/Nutrient Mixture F-12 (DMEM/F12; Thermo Fisher Scientific, Waltham, MA, USA; Cat. No. 11320-033) supplemented with 10% fetal bovine serum (FBS; American Type Culture Collection, Manassas, VA, USA), 100 U/mL penicillin–streptomycin solution (Antibiotic–Antimycotic solution (100×; Gibco, Thermo Fisher Scientific, Waltham, MA, USA), and 400 ng/mL hydrocortisone (Thermo Fisher Scientific, Waltham, MA, USA) for OSCC cell lines. All cell cultures were maintained under standard conditions in a humidified atmosphere containing 5% CO_2_ at 37 °C. The complete medium was replaced every second day. Upon reaching 70–80% confluence, cells were passaged.

### 2.2. Public Database Analysis

Data on expression of *IGF2*, *H19*, and their associated hsa-miR-483-3p, and hsa-miR-675-5p were obtained from The Cancer Genome Atlas (TCGA) HNSC cohort via Xena platform (https://xenabrowser.net/, accessed on 12 February 2026) [20]. Expression data in oral cancer and normal tissue were generated by RNASeq using the Illumina HiSeq platform (Illumina, Inc., San Diego, CA, USA). Dataset version 2017-10-13 was used for *IGF2* and *H19*, whereas dataset version 2017-09-08 was used for hsa-miR-483-3p, and hsa-miR-675-5p. Samples were filtered by anatomic neoplasm subdivision, and larynx, hypopharynx, oropharynx and tonsil were filtered out. The final dataset consisted of 347 (87.2%) primary tumor samples and 51 (12.8%) normal solid tissue samples. Data were downloaded for further statistical analysis. The total number of analyzed samples varied between candidates because expression data were missing for some samples.

### 2.3. Study Group and Biological Material

The study was conducted in accordance with the Declaration of Helsinki, and the protocol was approved by the Ethics Committee of the Faculty of Medicine of the University of Belgrade (approval number 1550/VII-6, 18 July 2019) and the Ethics Committee of the University Clinical Center of Serbia (approval number 1880/71, 25 December 2025). From all included patients, written informed consent was obtained. The study group consisted of 55 patients diagnosed with oral cancer. All patients underwent surgical treatment at the Clinic for Otorhinolaryngology and Maxillofacial Surgery, University Clinical Center of Serbia, between 2018 and 2024. All patients received adjuvant radiotherapy, while some also underwent adjuvant chemotherapy, depending on clinical indications. Demographic and clinical information of the study group is presented in Table 1. The median follow-up time was 17 months. Overall survival was defined as the time from oral cancer diagnosis to death from any cause and was presented in months. Data on overall survival time were unavailable for two patients who died and were therefore excluded from the survival analysis. Patients who were alive at the last follow-up were censored at that time.

During surgery, both tumor tissue and adjacent non-cancerous tissue (located at least 2 cm from the tumor edges) were collected. Non-cancerous tissue was histologically confirmed as non-malignant by the pathologist. Tissue samples were immediately preserved in RNAlater (Invitrogen, Waltham, MA, USA) and stored at −80 °C.

### 2.4. RNA Isolation

From cultured cell lines SCC-25, SCC-15 and HaCaT, total RNA was isolated by using TRIzol reagent (Invitrogen, Waltham, MA, USA) following manufacturer’s protocol. Total RNA was isolated from clinical samples by mirVana™ miRNA Isolation Kit (ThermoFisher Scientific, Waltham, MA, USA). Isolated RNA was stored at −80 °C. Concentration and purity of total RNA was measured at nanodrop (Implen, Munich, Germany). Relative expression of mRNAs and mature form of miRNAs was performed by qRT-PCR using the QuantStudio™ 3 Real-Time PCR System (Applied Biosystems, ThermoFisher Scientific, Waltham, MA, USA).

### 2.5. Reverse Transcription and Quantitative Real Time PCR (RT-qPCR)

For relative expression analysis of *IGF2* and *H19* gene, cDNA was synthesized by using High-Capacity cDNA Reverse Transcription Kit (Applied Biosystems, Thermo Fisher Scientific, Waltham, MA, USA) following the recommended protocol by the manufacturer. The reaction mix consisted of 10× RT buffer, 10× RT random primers, 25× dNTP mix, MultiScribe Reverse Transcriptase and 20ng of RNA in the final volume of 15 µL. To relatively quantify expression of miRNAs of our interest, cDNK was synthesized by using the TaqManTM microRNA reverse transcription kit (Thermo Fisher Scientific, Waltham, MA, USA) and pool of stem-loop primers following the manufacturer’s instructions. The thermal cycling conditions for cDNA synthesis were as follows: 30 min at 16 °C, 30 min at 42 °C, 5 min at 85 °C followed by a hold at 4 °C. All synthesized cDNAs were stored at −20 °C until qPCR.

Relative quantification of the expression levels of *IGF2*, *H19*, their hosted miRNAs, hsa-miR-483-3p and hsa-miR-675-5p, and endogenous controls *GAPDH* and *RNU6B* was performed using TaqMan^TM^ gene expression assays or TaqMan^TM^ microRNA gene expression assays (Thermo Fisher Scientific, Waltham, MA, USA): Hs00277496_s1 (*IGF2*), Hs00262142_g1 (*H19*), Hs99999905_m1 (*GAPDH*), ID 002339 (hsa-miR-483-3p), ID 002005 (hsa-miR-675-5p), ID 001093 (*RNU6B*). No AmpErase UNG Universal PCR Master Mix (Thermo Fisher Scientific, Waltham, MA, USA) was used in the qPCR reaction mix. Temperature profile was as follow: 5 min at 95 °C, followed by 40 cycles of 15 s at 95 °C and 1 min at 60 °C. All reactions were run in triplicate.

Relative expression of the mRNA, lncRNA and miRNAs were determined using the dCt method (ΔCt = Ct_sample_ − Ct_endogenous control_), and reported as 2^−ΔCt^. Results were presented as mean ± standard deviation (SD).

Experimental validation of both miRNAs in both cell lines and clinical samples was not feasible because of technical and resource constraints. Specifically, hsa-miR-483-3p was measured only in cell lines, whereas hsa-miR-675-5p was analyzed only in clinical samples. Results from the relative quantification of hsa-miR-675-5p expression have been previously published for a subgroup of 35 oral cancer patients [21], while in the current study, expression was evaluated in an enlarged group of patients (*n* = 48).

### 2.6. Statistical Analysis

Obtained data were analyzed using SPSS v22.00 (IBM SPSS Statistics, Armonk, NY, USA) and GraphPadPrism v9.0 software (GraphPadPrism software, Boston, MA, USA). Normality of data distribution was tested by Kolmogorov–Smirnov test and Shapiro–Wilk test. For normally distributed data, comparisons of relative expression were performed using the Student’s *t*-test. Data that showed not normal distribution were analyzed by non-parametric statistical Mann–Whitney test. Differences in relative expression of mRNAs and miRNAs between oral cancer and paired non-cancerous tissue were analyzed by Wilcoxon sign-rank test. Bonferroni correction was applied for multiple testing. Correlation between relative expression levels of *IGF2*, *H19* and their hosted miRNAs was assessed using Pearson’s correlation test for normally distributed data and Spearman’s correlation test for non-normally distributed data. Associations between expression and clinical characteristics were determined by the χ^2^ test. Discriminatory potential was estimated by Receiver operating curve (ROC) with Area under the curve (AUC) and 95% confidence interval (95% CI). Optimal cut-off value was determined by maximal Youden index, and for cut-off value specificity and sensitivity were calculated. Positive predictive value (PPV) and negative predictive value (NPV) were calculated based on the sensitivity and specificity values obtained at the optimal cut-off. Given the design of the current study with an equal number of cancer and non-cancerous samples (1:1 ratio), the calculated PPV and NPV reflect the diagnostic performance within the study cohort and do not represent population-based predictive values based on disease prevalence. Predicted probability from the logistic regression model based on combination of *IGF2* and *H19* to distinguish between oral cancer and non-cancerous tissue was calculated as follows: logit(p) = 0.143 + (−0.259 × 2^−ΔCt *IGF2*^) + (−0.064 × 2^−ΔCt *H19*^); combination of *IGF2*, *H19* and hsa-miR-675-5p was calculated as follows: logit(p) = 0.540 + (−0.308 × 2^−ΔCt *IGF2*^) + (−0.060 × 2^−ΔCt *H19*^) + (−1.255 × 2^−ΔCt hsa-miR-675-5p^). Oral cancer patients were dichotomized according to the expression of *IGF2*, *H19* and hsa-miR-675-5p in cancer tissue into low and high expressed group based on median of expression. Overall survival between patients with low and high expression was compared by log-rank test of the Kaplan–Meier survival curves. Hazard ratio for outcome was calculated by Cox proportional regression analysis with 95% CI. Power of the study was calculated by G*Power 3.1 software [22]. *p* values were two-tailed and if less than 0.05 were considered significant.

## 3. Results

### 3.1. Expression Analysis of IGF2, H19 and hsa-miR-483-3p in Oral Cancer Cell Lines

**Figure 1:**
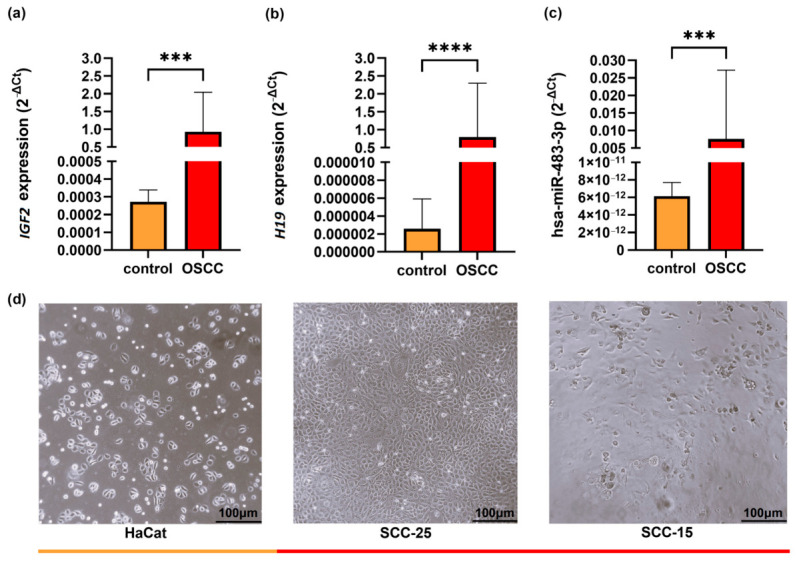
Expression profile of *IGF2* (**a**), *H19* (**b**) and hsa-miR-483-3p (**c**) in oral cancer (OSCC: SCC-25, SCC-15) and control cell line (HaCaT). Representative micrographs of healthy keratinocytes and OSCC cell lines, acquired using an inverted light microscope at 10× magnification, are shown in panel (**d**). Data are presented as mean ± standard deviation (SD). Statistical significance is shown with symbols: *** *p* < 0.001, **** *p* < 0.0001.

Expression levels of *IGF2*, *H19* and hsa-miR-483-3p were evaluated in oral cancer cell lines SCC-25 and SCC-15 (Figure 1). Compared with the control HaCaT cell line, both *IGF2* and *H19* showed significantly elevated expression levels in the OSCC cell lines (*p* = 0.0003, *p* < 0.0003, respectively, Mann–Whitney test, Bonferroni correction). Similarly, hsa-miR-483-3p expression was significantly upregulated in the oral cancer cell lines compared to the control (*p* = 0.0003, Mann–Whitney test, Bonferroni correction).

### 3.2. Public Database Analysis of IGF2, H19, hsa-miR-483-3p and hsa-miR-675-5p Expression and Its Biomarker Potential

**Figure 2:**
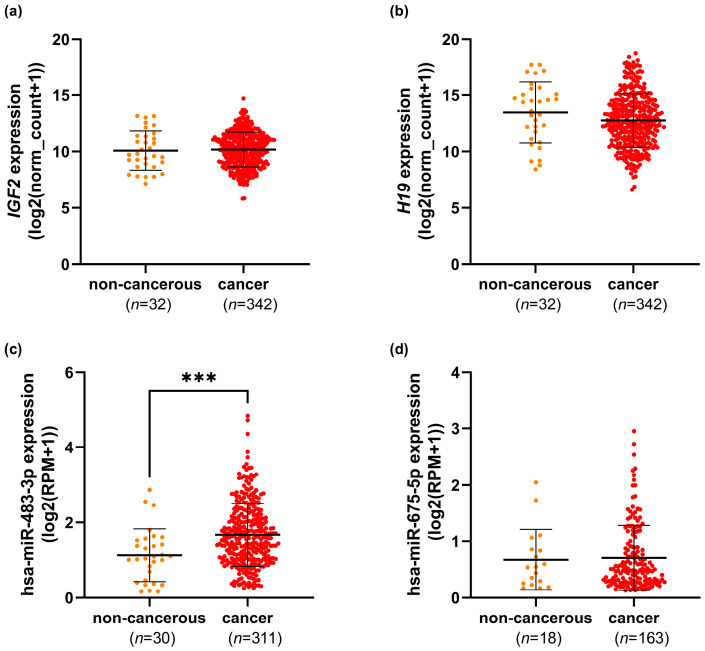
Expression profile of *IGF2* (**a**), *H19* (**b**), hsa-miR-483-3p (**c**) and hsa-miR-675-5p (**d**) in oral cancer and non-cancerous tissue, retrieved from the TCGA-HNSC dataset. Data are presented as mean ± standard deviation (SD). Statistical significance is shown with symbol: *** *p* < 0.001.

To evaluate expression of *IGF2*, *H19*, hsa-miR-483-3p and hsa-miR-675-5p in oral cancer and normal tissue, we performed analysis of the public TCGA-HNSC dataset (Figure 2). *IGF2* was slightly elevated in tumor but without significance compared to normal tissue (*p* = 1.000, *t*-test, Welch’s and Bonferroni correction). There was no significant difference in expression level of *H19* and hsa-miR-675-5p in tumor compared to normal tissue (*p* = 0.588; *p* = 1.000, respectively, *t*-test, Welch’s and Bonferroni correction). Level of hsa-miR-483-3p was significantly upregulated in cancer tissue (*p* = 0.0008, *t*-test, Welch’s and Bonferroni correction).

When paired samples from the TCGA-HNSC dataset were analyzed, a consistent expression trend was observed for *IGF2*, *H19*, hsa-miR-483-3p and hsa-miR-675-5p (Supplementary Figure S1). However, a statistically significant difference between cancer and paired non-cancerous tissues was detected only for hsa-miR-483-3p, which was significantly upregulated in cancer tissue compared with non-cancerous tissue (*p* = 0.008, *t*-test, Bonferroni correction).

In the same dataset, significant but moderate correlation in *IGF2* and *H19* expression in cancer tissue was observed (Pearson’s rho = 0.204, *p* = 0.0001), while strong correlation between *H19* and hsa-miR-675-5p was observed (Spearman’s rank rho = 0.653, *p* < 0.0001). There was also significant correlation of IGF2 and hsa-miR-483-3p expression in cancer tissue (Spearman’s rank rho = 0.512, *p* < 0.0001), Supplementary Figure S2.

**Figure 3:**
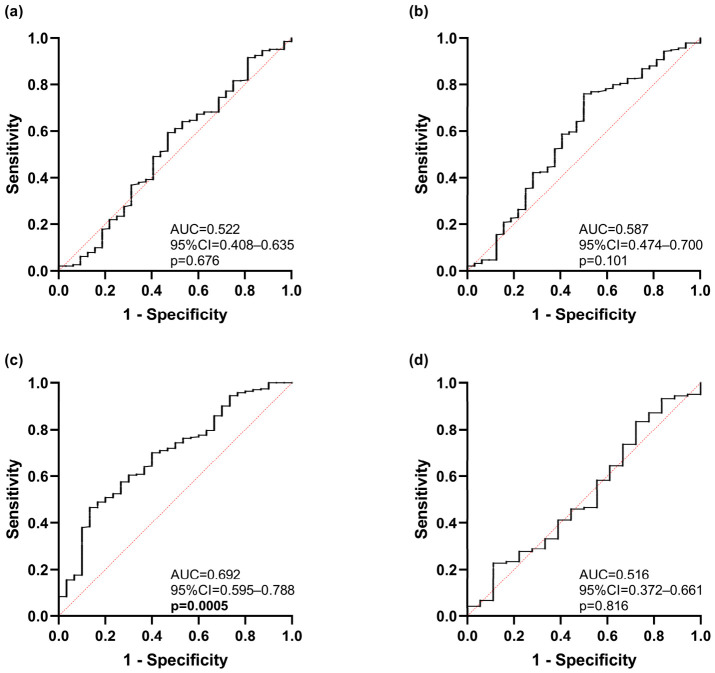
*IGF2* (**a**), *H19* (**b**), hsa-miR-483-3p (**c**) and hsa-miR-675-5p (**d**) potential to discriminate between oral cancer and non-cancerous tissue. ROC analysis was performed on data retrieved from the TCGA-HNSC dataset. The red line indicates reference.

Potential of *IGF2*, *H19*, hsa-miR-483-3p and hsa-miR-675-5p to discriminate between oral cancer and non-cancerous tissue was estimated by ROC analysis (Figure 3). Among the analyzed candidates, only hsa-miR-483-3p demonstrated moderate discriminatory potential as a biomarker (AUC = 0.692, 95% CI = 0.595–0.788, *p* = 0.0005), whereas no significant discriminatory performance was observed for *IGF2*, *H19* and hsa-miR-675-5p.

None of the analyzed *IGF2*, hsa-miR-675-5p and hsa-miR-483-3p were associated with overall survival according to Kaplan–Meier analysis and therefore do not appear to have prognostic value (Supplementary Figure S3). However, there was a significant difference in overall survival between patients with low and high *H19* expression in cancer tissue (*p* = 0.010, log-rank test). Patients with low *H19* expression had worse overall survival.

### 3.3. Expression Analysis of IGF2, H19 and hsa-miR-675-5p in Oral Cancer Clinical Samples and Association with Clinicopathological Characteristics

**Figure 4:**
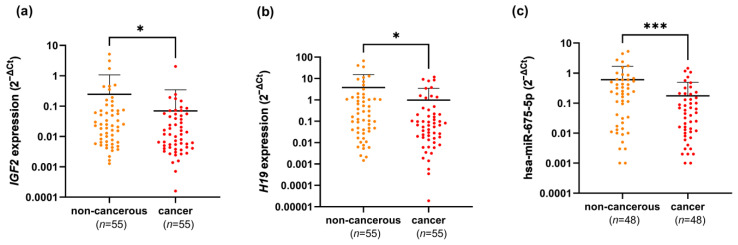
Expression profile of *IGF2* (**a**), *H19* (**b**) and hsa-miR-675-5p (**c**) in oral cancer and adjacent non-cancerous tissue. Data are presented as mean ± standard deviation (SD). The y-axis is on log10 scale. Statistical significance is shown with symbols: * *p* < 0.05, *** *p* < 0.001.

The expression profiles of *IGF2*, *H19*, and hsa-miR-675-5p in clinical samples from oral cancer patients are presented in Figure 4 and Supplementary Figure S4. A significant difference in relative expression between oral cancer tissue and adjacent non-cancerous tissues was observed for *IGF2* (*p* = 0.021, Wilcoxon signed-rank test, Bonferroni correction) and *H19* (*p* = 0.039, Wilcoxon signed-rank test, Bonferroni correction). Both *IGF2* and *H19* showed significantly decreased expression in oral cancer tissues compared to non-cancerous tissues, exhibiting approximately 3.5-fold and 3.9-fold lower expression levels, respectively. Similarly, hsa-miR-675-5p—which is transcribed from *H19*—was also downregulated in oral cancer compared to adjacent non-cancerous tissue (*p* < 0.0003, Wilcoxon signed-rank test, Bonferroni correction), showing 3.4-fold decrease in expression. Based on 55 paired oral cancer and non-cancerous tissue samples, the post hoc calculated power of the study was 0.99, assuming an effect size of 0.8 and a significance level of alpha 0.05.

Strong positive correlation was noticed between *IGF2* and *H19* expression in oral cancer tissue (Spearman’s rho = 0.601, *p* < 0.0001). In group of 48 clinical samples, there was weak, non-significant negative correlation between *H19* and hsa-miR-675-5p expression in oral cancer tissue (Spearman’s rho= −0.013, *p* = 0.929), Supplementary Figure S5.

Association of *IGF2*, *H19* and hsa-miR-675-5p expression in tumor tissue with demographic and clinicopathological characteristics of oral cancer patients is presented in Table 2. Recurrences were associated with *H19* expression (*p* = 0.022, χ^2^ test). No other significant associations were observed with clinicopathological characteristics.

### 3.4. Biomarker Potential of IGF2, H19 and hsa-miR-675-5p in Oral Cancer

**Figure 5:**
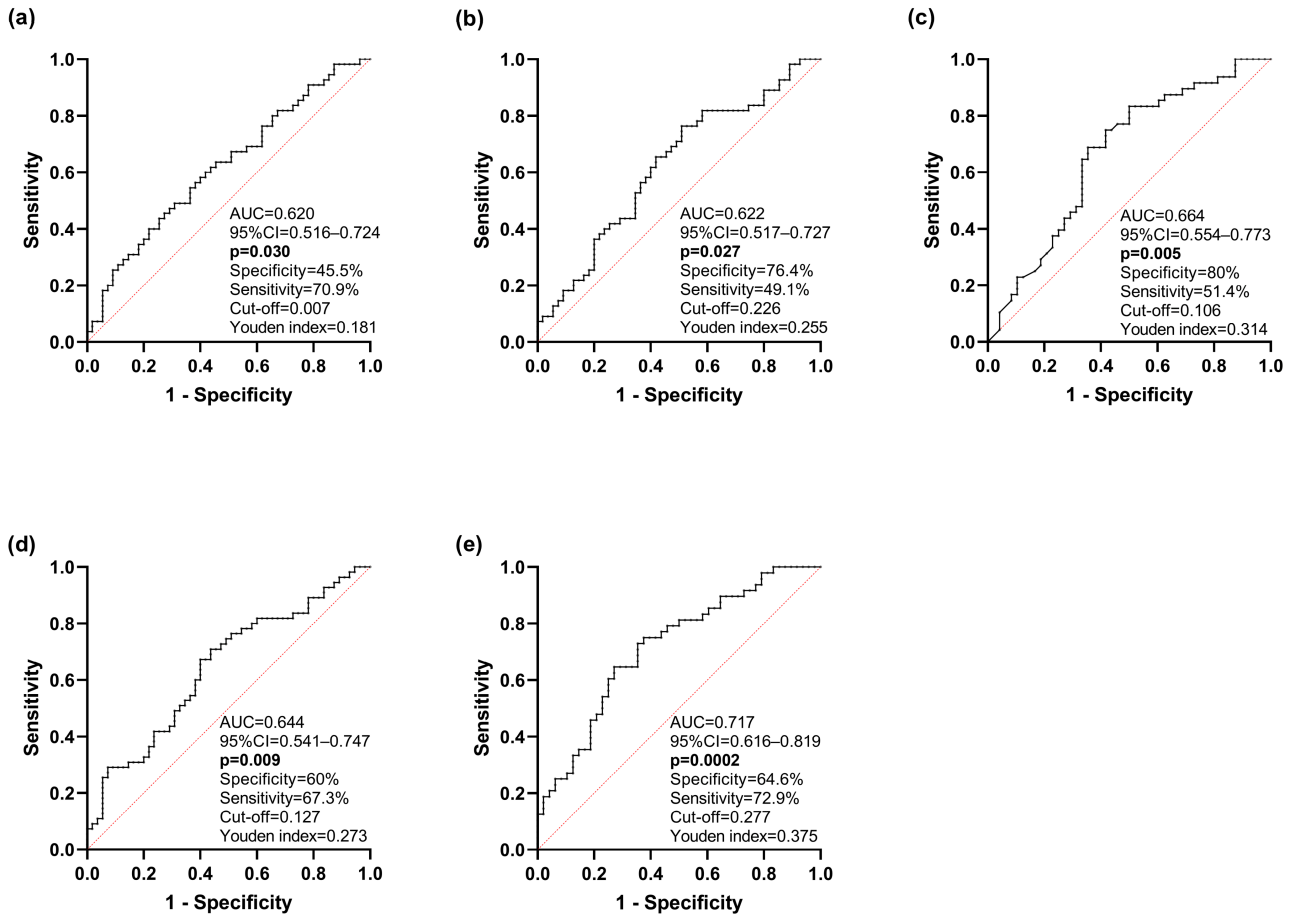
(**a**) IGF2 (**b**) H19 (**c**) hsa-miR-675-5p (**d**) combined IGF2 and H19 (**e**) combined IGF2, H19 and hsa-miR-675-5p potential to discriminate between oral cancer and adjacent non-cancerous tissue. The red line indicates reference.

Figure 5 presents ROC curves demonstrating the discriminatory performance of the analyzed candidates. Both *IGF2* and *H19* showed moderate, yet promising ability to discriminate between oral cancer and non-cancerous tissue according to results of ROC analysis (AUC = 0.620, 95% CI = 0.516–0.724, *p* = 0.030, PPV = 56.5%, NPV = 61%; AUC = 0.622, 95% CI = 0.517–0.727, *p* = 0.027, PPV = 67.5%, NPV = 60%, respectively). The relative expression of hsa-miR-675-5p also showed moderate discriminatory potential for distinguishing oral cancer from non-cancerous tissue (AUC = 0.664, 95% CI = 0.554–0.773, *p* = 0.005, PPV = 72%, NPV = 62.2%). The combined analysis of *IGF2* and *H19* improved discriminatory performance compared to individual biomarkers (AUC = 0.644, 95% CI = 0.541–0.747, *p* = 0.009, PPV = 62.7%, NPV = 64.7%). Importantly, the integration of all three analyzed molecules further enhanced the ability to discriminate between oral cancer and non-cancerous tissue, yielding the highest test performance (AUC = 0.717, 95% CI = 0.616–0.819, *p* = 0.0002, PPV = 67.3%, NPV = 70.4%). PPV and NPV values reflect test performance within the study cohort.

**Figure 6:**
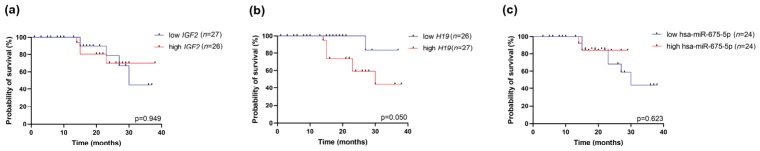
Kaplan–Meier curves of overall survival in oral cancer patients depending on *IGF2* (**a**), *H19* (**b**), and hsa-miR-675-5p (**c**) expression levels. Low and high levels of relative expression refer to values above or below the median. Survival data were missing for two patients; therefore, they were excluded from the survival analysis.

To evaluate the prognostic potential of *IGF2*, *H19*, and hsa-miR-675-5p, Kaplan–Meier survival analysis was performed, and survival curves for low- and high-expression of analyzed candidates were compared (Figure 6). No significant differences in overall survival were observed between patients with low and high expression levels of *IGF2*, *H19*, or hsa-miR-675-5p in oral cancer tissue.

*IGF2*, *H19* and hsa-miR-675-5p cannot be used as independent predictors of mortality in oral cancer patients based on the results of the univariate and multivariate Cox proportional hazard ratio analysis (Table 3).

## 4. Discussion

The purpose of this study was to perform a comprehensive analysis of the relative expression of *IGF2*, *H19*, and their hosted miRNAs, hsa-miR-483-3p and hsa-miR-675-5p, originating from the *IGF2-H19* locus, in oral cancer cell lines, publicly available datasets and clinical samples. The use of three independent study models indicates the strength of the present study. Cell lines provided a controlled experimental system that enabled the evaluation of gene expression in a homogeneous tumor cell population without the influence of the tumor microenvironment. Publicly available datasets allowed expression evaluation in a larger cohort of patients, while our clinical samples reflected the biological complexity and heterogeneity of OSCC in vivo. The differences observed among these models should not necessarily be considered contradictory; rather, they highlight the complexity of *IGF2-H19* regulation and the influence of different biological contexts. Together, these complementary approaches provide a more comprehensive understanding of gene expression patterns than any single model alone.

We first evaluated the expression of *IGF2*, *H19*, and hsa-miR-483-3p in commercially available OSCC cell lines and healthy keratinocytes. All three molecules tested in cell lines were significantly upregulated in OSCC compared with control cells, suggesting coordinated deregulation of transcripts originating from the *IGF2-H19* locus in oral cancer. Based on our findings in OSCC cell lines, both *IGF2*, *H19* and hsa-miR-483-3p may be considered to have protooncogenic roles. These results are consistent with previous studies reporting the increased level of *IGF2* [10] and *H19* in oral cancer cell lines [23,24]. It has been demonstrated that *H19* promotes metastasis and invasion in tongue cancer cells through the let-7/HMGA2 axis [23]. Furthermore, another study suggested that the increased expression of *H19* in oral cancer cell lines is associated with hypomethylation of the *H19* promoter [13] indicating the importance of consideration of methylation level.

To further validate and expand these findings, we analyzed publicly available datasets containing gene expression profiles from healthy and tumor oral tissue samples.

Contrary to our observations in cell lines, a statistically significant difference between cancer and non-cancerous tissues was observed only for hsa-miR-483-3p, whereas *IGF2* and *H19* expression levels did not differ significantly in the TCGA-HNSC cohort. These unexpected findings indicated that the expression patterns observed in established cell lines may not fully reflect those present in clinical specimens. Although hsa-miR-483 is located within the *IGF2* genomic locus and generates two mature transcripts—miR-483-5p and miR-483-3p—evidence suggests that its transcription is not solely dependent on *IGF2* expression [25]. Consequently, we expanded our investigation by including hsa-miR-675-5p, another microRNA transcribed from the *IGF2-H19* locus. Similar to *IGF2* and *H19*, TCGA-HNSC dataset analysis did not reveal statistically significant differences in hsa-miR-675-5p expression between healthy and tumor tissues. Given these inconclusive results and the observed discrepancies between in vitro cell line models and publicly available tissue datasets, we decided to evaluate the expression of *IGF2*, *H19*, and hsa-miR-675-5p in our own cohort of clinical OSCC specimens in order to better define their potential involvement in oral carcinogenesis and their value as diagnostic and prognostic biomarkers.

In our study group, *IGF2* was significantly decreased as *H19* and hsa-miR-675-5p in oral cancer clinical samples. The differences in *IGF2* and *H19* expression profiles observed between oral cancer cell lines and clinical samples may be attributed to differences in cellular composition, tumor heterogeneity and microenvironment. Cell lines consist exclusively of tumor cells and lack stromal and infiltrating cell populations, whereas expression analysis in clinical samples reflects bulk tissue expression. Therefore, cell lines may not accurately represent the expression profiles observed in clinical specimens. Furthermore, there were differences in *IGF2* expression between TCGA-HNSC dataset analysis and our clinical samples. Although the expression trends for *H19* and hsa-miR-675-5p were in line with our findings in clinical samples, the TCGA-HNSC dataset analysis did not reveal statistically significant differences in expression between oral cancer and normal tissue samples. This inconsistency may be attributed to differences in methodology, including the use of RT-qPCR in our study and RNA sequencing in the TCGA-HNSC dataset, as well as differences in sample size. Additionally, our study utilized adjacent non-cancerous tissue as a control, which may have influenced the results due to the potential presence of a field of cancerization [26,27,28].

Previous research found no differences in *IGF2* expression between normal and cancer tissues in patients with head and neck squamous cell carcinoma, including tumors located in the larynx, pharynx, or oral cavity [29]. The discrepancy between the previous report and our findings may reflect differences in study group, sample size, tumor localization, disease stage, and experimental methodology, all of which could influence *IGF2* expression patterns.

Numerous reports showed deregulation of *H19* in OSCC. However, data are contradictory, as *H19* was identified as both downregulated and upregulated in oral cancer, as reported in recent systematic review [30]. *H19* level was significantly decreased in oral cancer tissue compared to non-cancerous tissue in our study which was in line with earlier reported findings [12,31,32]. On the contrary, *H19* was increased in tongue cancer tissue compared to adjacent normal tissue, confirming its oncogenic role [23,24]. *H19* and hsa-miR-675-5p were reported as upregulated in oral cancer tissue compared to normal tissue from unrelated healthy individuals [30]. In clinical samples of head and neck squamous cell carcinoma—including cancers of the larynx, oropharynx, and hypopharynx—*H19* and hsa-miR-675 were reported to be upregulated in tumor tissue compared with noncancerous tissue, and this finding was associated with prognostic significance [33]. These findings contrast to our results, which were limited to oral cavity tumor sites. This discrepancy underscores the importance of considering anatomical sites-specific differences and highlights the heterogeneity of cancers arising within the head and neck region.

*H19* and its associated hsa-miR-675-5p show context-dependent expression profile and oncogenic or tumor suppressor function across different cancer types. For instance, *H19* expression was increased in tumor tissue of patients with locally advanced rectal carcinoma [34]. Opposite findings for different tumor types, even though all are developed of the epithelial cells, can be explained by context-dependent specificity, as it was shown that *H19* behaves as protooncogene, as well as tumor suppressor gene in different tumor tissues. Furthermore, different methylation patterns, loss of imprinting, tumor environment, but also technical and methodological factors might contribute to disparities among different cancer types.

While studies performed on cell lines generally report increased expression of these genes, analyses of tissue samples have produced inconsistent findings, resulting in conflicting data in the literature and raising questions regarding the causes of these discrepancies [32,35]. The results obtained from cell lines reflect strictly controlled experimental conditions and the absence of the tumor microenvironment. Furthermore, OSCC cell lines represent well-established in vitro models derived from aggressive oral squamous cell carcinomas and are capable of long-term propagation under controlled culture conditions. Therefore, their molecular profile may not fully correspond to that observed in clinical specimens. Future studies may benefit from incorporating data obtained from primary cell cultures, which could provide an intermediate model between established cell lines and patient-derived tissues.

One of the aims of our study was to evaluate translational potential of *IGF2*, *H19*, and hsa-miR-675-5p expression as biomarkers for the discrimination of cancer and non-cancerous tissues. We have shown that the combination of *IGF2*, *H19*, and hsa-miR-675-5p yields the highest test performance for distinguishing oral cancer from non-cancerous tissue. The superior performance of the combined model supports the concept that simultaneous assessment of multiple components of the *IGF2-H19* locus may provide complementary diagnostic information and better reflect locus dysregulation than individual biomarkers. The *IGF2*, *H19* and hsa-miR-675-5p biomarker profile could serve as a promising valuable tool to conventional histopathology, potentially aiding in the more precise definition of surgical margins. Also, these findings might potentially contribute to the development of sensitive diagnostic tools necessary to overcome the challenges of late-stage oral cancer detection. Nevertheless, these findings require validation in larger independent cohorts before any conclusions regarding clinical utility can be drawn.

None of the analyzed candidates was considered a potential prognostic biomarker. Results from previous studies on this topic are also conflicting. One previous study identified *H19* as significant prognostic biomarker for both overall survival and relapse [34]. The absence of a significant association with overall survival should be interpreted with caution, as the relatively limited sample size and restriction of the cohort to oral cavity tumors may have reduced the power to detect prognostic effects. Additionally, the low number of death events observed during follow-up may have reduced the statistical power of the survival analyses and hindered the detection of potentially relevant prognostic associations. Therefore, the negative findings reported herein should not be regarded as conclusive evidence that these biomarkers lack prognostic relevance. The discrepancy between the TCGA-HNSC and clinical cohort findings regarding the prognostic significance of *H19* remains unclear; however, differences in clinicopathological characteristics, treatment strategies, follow-up duration, and analytical methodologies between the cohorts, together with the limited number of death events observed in our study, may have contributed to the divergent findings.

When interpreting results from the current study, it is important to acknowledge several limitations. The limitation of this study is the use of only two OSCC cell lines—SCC-15 and SCC-25, which may not fully capture the biological heterogeneity of oral squamous cell carcinoma. In addition, HaCaT cells were used as the control group; however, as immortalized epidermal keratinocytes, they do not represent a true equivalent of normal oral keratinocytes. Therefore, the results obtained from cell line experiments should be interpreted with these limitations in mind. Furthermore, we used only one endogenous control—*GAPDH* for mRNA and lncRNA and *RNU6B* for miRNAs. Therefore, this limitation should be considered when interpreting the diagnostic and prognostic potential of *H19*, *IGF2*, and hsa-miR-675-5p. hsa-miR-483-3p and hsa-miR-675-5p were not experimentally validated in both cell lines and clinical samples due to technical and resource constraints. The incomplete validation of both miRNAs across all experimental settings limits our ability to draw firm conclusions regarding the coordinated regulation of the *IGF2-H19* locus and should therefore be considered when interpreting the findings. Nevertheless, integration of experimental findings with a publicly available dataset provided additional support for the observed expression profile of *IGF2-H19* locus. Although we had a relatively small number of clinical samples, we analyzed data from the TCGA-HNSC project using the Xena platform. Our patient group was limited in size, but it remains a valuable resource, as the number of paired samples was greater compared to the TCGA dataset. Despite the relatively limited sample size, our study group was sufficient to detect differences in *IGF2*, *H19*, and hsa-miR-675-5p expression between cancerous and non-cancerous tissues, assuming an effect size of 0.8, statistical power of 0.8, and an alpha level of 0.05.

## 5. Conclusions

The results of the present study suggest that genes within the *IGF2-H19* locus, together with the associated hsa-miR-675-5p expression, may serve as potential molecular biomarkers for sensitive discrimination of oral cancer from non-cancerous tissue. Diagnostic performance was further improved when the expression levels of *IGF2*, *H19*, and hsa-miR-675-5p were analyzed in combination. However, validation in larger, independent patient cohorts is required to confirm these findings and to further elucidate the role of *IGF2-H19* locus gene expression in oral cancer prior to potential clinical implication. In addition, mechanistic studies using advanced experimental models, including 3D culture systems, are warranted to investigate the biological functions of these genes and the epigenetic regulation of the *IGF2-H19* locus in oral cancer.

## Figures and Tables

**Table 1:** Characteristics of the study group.

Characteristics		*n*	%
Sex	male	42	76.4
	female	13	23.6
Age (years)	mean ± SD	61.44 ± 9.729	
Age (median)	≤55 years	14	25.5
	>55 years	41	74.5
Alcohol use	no intake	23	41.8
	moderate	19	34.5
	high	13	23.6
Smoking status	non-smoker	14	25.5
	ex-smoker	26	47.3
	smoker	15	27.3
Oral hygiene	good	17	30.9
	poor	38	69.1
Location of the primary tumor	tongue	30	54.5
	floor of the mouth	15	27.3
	hard palate	5	9.1
	lower jaw mucosa	2	3.6
	upper jaw mucosa	1	1.8
	buccal mucosa	2	3.6
Histological grade	well differentiated	14	25.5
	moderately differentiated	30	54.5
	poorly differentiated	11	20
TNM stage	I	4	7.3
	II	19	34.5
	III	10	18.2
	IV	22	40
Tumor size	<2 cm	4	7.3
	2–4 cm	22	40
	>4 cm	22	40
	infiltrated	7	12.7
Lymph-node status	N0	29	52.7
	N1	5	9.1
	N2	6	10.9
	N3	15	27.3
Distant metastases	no	55	100
	yes	0	0
Recurrence	no	44	80
	yes	11	20
Disease outcome	alive	44	80
	death	11	20

SD—standard deviation.

**Table 2:** Association of *IGF2-H19* and hsa-miR-675-5p expression with demographic and clinicopathological characteristics of oral cancer patients.

		Relative Expression in Oral Cancer Tissue								
Demographic and Clinicopathological Characteristics		*IGF2*			*H19*			hsa-miR-675-5p		
		Low (*n* = 28)	High (*n* = 27)	*p*	Low (*n* = 27)	High (*n* = 28)	*p*	Low (*n* = 24)	High (*n* = 24)	*p*
Sex	male	25 (89.3)	17 (63)	0.022	24 (88.9)	18 (64.3)	0.032	18 (75)	19 (79.2)	0.731
	female	3 (10.7)	10 (37)		3 (11.1)	10 (35.7)		6 (25)	5 (20.8)	
Age (years, median)	<55	7 (25)	7 (25.9)	0.937	8 (29.6)	6 (21.4)	0.485	5 (20.8)	7 (29.2)	0.505
	>55	21 (75)	20 (74.1)		19 (70.4)	22 (78.6)		19 (79.2)	17 (70.8)	
Smoking habits	nonsmoker	5 (17.9)	10 (37)	0.110	5 (18.5)	10 (35.7)	0.152	6 (25)	7 (29.2)	0.745
	smoker + ex-smoker	23 (82.1)	17 (63)		22 (81.5)	18 (64.3)		18 (75)	17 (70.8)	
Alcohol consumption	no intake	12 (42.9)	11 (40.7)	0.874	12 (44.4)	11 (39.3)	0.698	9 (37.5)	10 (41.7)	0.768
	moderate + high	16 (57.1)	16 (59.3)		15 (55.6)	17 (60.7)		15 (62.5)	14 (58.3)	
Oral hygiene	good	7 (25)	10 (37)	0.334	7 (25.9)	10 (35.7)	0.432	9 (37.5)	7 (29.2)	0.540
	poor	21 (75)	17 (63)		20 (74.1)	18 (64.3)		15 (62.5)	17 (70.8)	
Location of primary tumor	tongue	12 (42.9)	18 (66.7)	0.301	12 (44.4)	18 (64.3)	0.418	15 (62.5)	14 (58.3)	0.144
	hard palate	4 (14.3)	1 (3.7)		2 (7.4)	3 (10.7)		4 (16.7)	0 (0)	
	floor of the mouth	8 (28.6)	7 (25.9)		9 (33.3)	6 (21.4)		4 (16.7)	7 (29.2)	
	lower jaw mucosa	2 (7.1)	0 (0)		2 (7.4)	0 (0)		0 (0)	2 (8.3)	
	upper jaw mucosa	1 (3.6)	0 (0)		1 (3.7)	0 (0)		-	-	
	buccal swab	1 (3.6)	1 (3.7)		1 (3.7)	1 (3.6)		1 (4.2)	1 (4.2)	
Histological gradus	well differentiated	8 (28.6)	6 (22.2)	0.445	7 (25.9)	7 (25)	0.902	7 (29.2)	6 (25)	0.634
	moderately differentiated	13 (46.4)	17 (63)		14 (51.9)	16 (57.1)		12 (50)	15 (62.5)	
	poorly differentiated	7 (25)	4 (14.8)		6 (22.2)	5 (17.9)		5 (20.8)	3 (12.5)	
Stage	I + II	10 (35.7)	13 (48.1)	0.350	12 (44.4)	11 (39.3)	0.698	9 (37.5)	10 (41.7)	0.768
	III + IV	18 (64.3)	14 (51.9)		15 (55.6)	17 (60.7)		15 (62.5)	14 (58.3)	
Tumor size	≤2 cm	0 (0)	4 (14.8)	0.093	0 (0)	4 (14.3)	0.093	2 (8.3)	1 (4.2)	0.763
	2–4 cm	13 (46.4)	9 (33.3)		13 (48.1)	9 (32.1)		10 (41.7)	9 (37.5)	
	>4 cm	15 (53.6)	14 (51.9)		14 (51.9)	15 (53.6)		12 (50)	14 (58.3)	
Nodal status	N−	13 (46.4)	16 (59.3)	0.341	14 (51.9)	15 (53.6)	0.898	10 (41.7)	16 (66.7)	0.082
	N+	15 (53.6)	11 (40.7)		13 (48.1)	13 (46.4)		14 (58.3)	8 (33.3)	
Recurrences	no	22 (78.6)	22 (81.5)	0.787	25 (92.6)	19 (67.9)	0.022	17 (70.8)	22 (91.7)	0.064
	yes	6 (21.4)	5 (18.5)		2 (7.4)	9 (32.1)		7 (29.2)	2 (8.3)	

**Table 3:** Impact of *IGF2*, *H19* and hsa-miR-675-5p expression on overall survival outcomes in oral cancer patients.

	Overall Survival	
Univariate Analysis	HR (95% CI)	*p*
*IGF2*	0.193 (0.000–305.011)	0.662
*H19*	0.969 (0.751–1.250)	0.808
hsa-miR-675-5p	0.847 (0.020–36.509)	0.931
Multivariate analysis		
*IGF2*	0.237 (0.000–220.297)	0.680
*H19*	0.996 (0.754–1.316)	0.978
hsa-miR-675-5p	0.817 (0.019–34.955)	0.916

HR (95% CI)—Hazard ratio with 95% confidence interval.

## Data Availability

The data presented in this study are available upon request from the corresponding author.

## Supplementary Materials

The following supporting information can be downloaded at: https://www.mdpi.com/article/10.3390/biology15151221/s1, Figure S1: Expression profile of *IGF2* (a), *H19* (b), hsa-miR-483-3p (c) and hsa-miR-675-5p (d) in oral cancer and non-cancerous tissue, retrieved from the TCGA-HNSC dataset. Data are presented as mean ± standard deviation (SD). Statistical significance is shown with symbol: ** *p* < 0.01; Figure S2: Correlation analysis between expression levels of *IGF2* and *H19* (a) *IGF2* and its hosted hsa-miR-483-3p (b), and *H19* and its hosted hsa-miR-675-5p (c). Correlation analysis was done for expression data in oral cancer tissue. Data were retrieved from the TCGA-HNSC dataset.; Figure S3: Kaplan–Meier curves of overall survival in oral cancer patients depending on *IGF2* (a), *H19* (b), hsa-miR-483-3p (c), and hsa-miR-675-5p (d) expression levels. All data were retrieved from the TCGA-HNSC dataset. Low and high levels of relative expression refer to values above or below the median; Figure S4: Expression profile of *IGF2* (a), *H19* (b) and hsa-miR-675-5p (c) in oral cancer and adjacent non-cancerous tissue—paired samples plot. The y-axis is on log10 scale. Statistical significance is shown with symbols: * *p* < 0.05, ** *p* < 0.01, *** *p* < 0.001; Figure S5: Correlation analysis between expression levels of *IGF2* and *H19* (a), and *H19* and its hosted hsa-miR-675-5p (b) in oral cancer clinical samples from our study group.

## Abbreviations

The following abbreviations are used in this manuscript:

95% CI	95% confidence interval
AUC	Area under the curve
DMEM/F12	Dulbecco’s Modified Eagle Medium/Nutrient Mixture F-12
FBS	fetal bovine serum
HNSC	Head and neck squamous cell carcinoma
HR	Hazard ratio
lncRNA	long noncoding RNA
miRNAs	microRNAs
mRNA	Messenger RNA
NPV	Negative predictive value
OSCC	Oral squamous cell carcinoma
PPV	Positive predictive value
ROC	Receiver operating curve
SD	Standard deviation
TCGA-HNSC	The Cancer Genome Atlas—Head and Neck Squamous Cancer
TNM	Tumor, node, metastasis
