# Supplementary material for: IGF2-H19 Locus Expression Profile and Biomarker Potential in Oral Cancer
Source: Biology (Basel). 2026 Jul 23;15(15):1221. doi: 10.3390/biology15151221 (PMC13465723; doi:10.3390/biology15151221)
Supplement: Supplementary file 1 [file biology-15-01221-s001.zip › biology-4382943-supplementary.pdf]

Article

***IGF2-H19* locus expression profile and biomarker potential in oral cancer**

Supplementary material

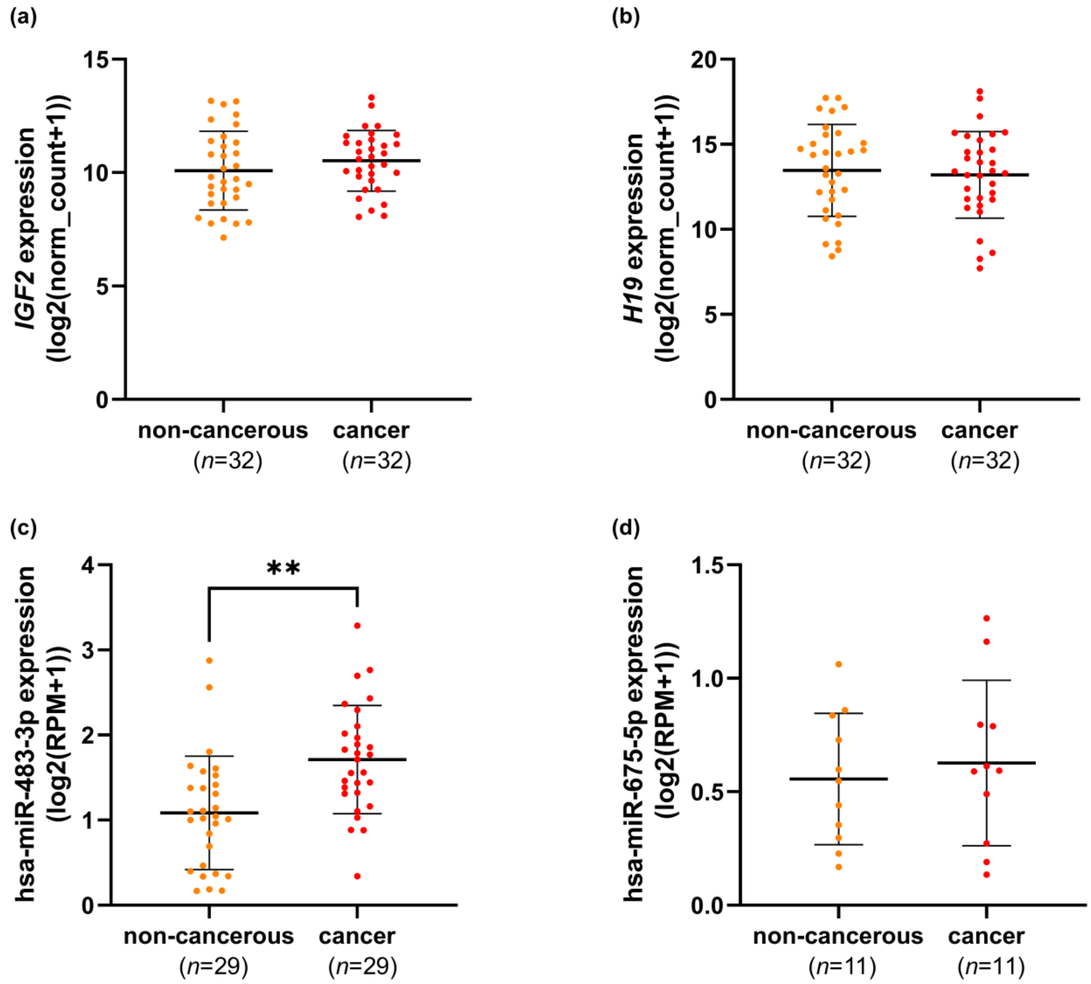

Figure S1. Expression profile of *IGF2* (a), *H19* (b), hsa-miR-483-3p (c) and hsa-miR-675-5p (d) in oral cancer and non-cancerous tissue, retrieved from the TCGA-HNSC dataset. Data are presented as mean ± standard deviation (SD). Statistical significance is shown with symbol: **p<0.01.

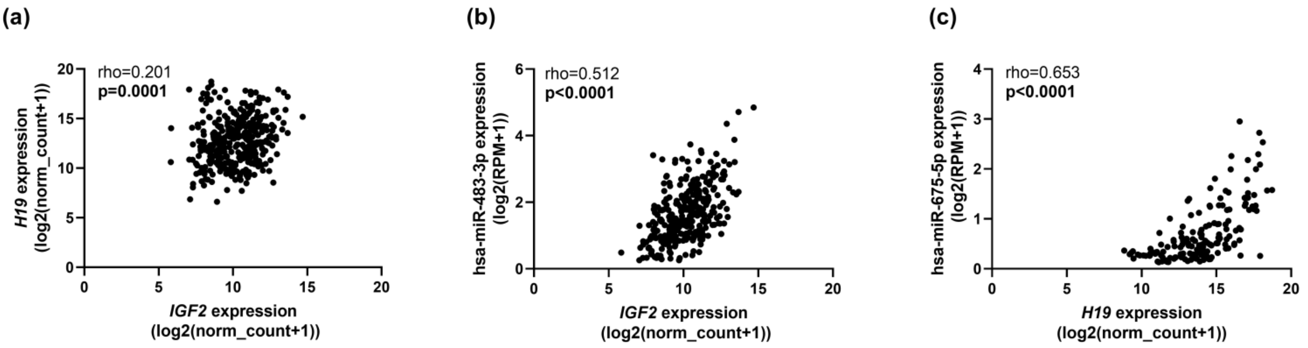

Figure S2. Correlation analysis between expression levels of *IGF2* and *H19* (a) *IGF2* and its hosted hsa-miR-483-3p (b), and *H19* and its hosted hsa-miR-675-5p (c). Correlation analysis was done for expression data in oral cancer tissue. Data were retrieved from the TCGA-HNSC dataset.

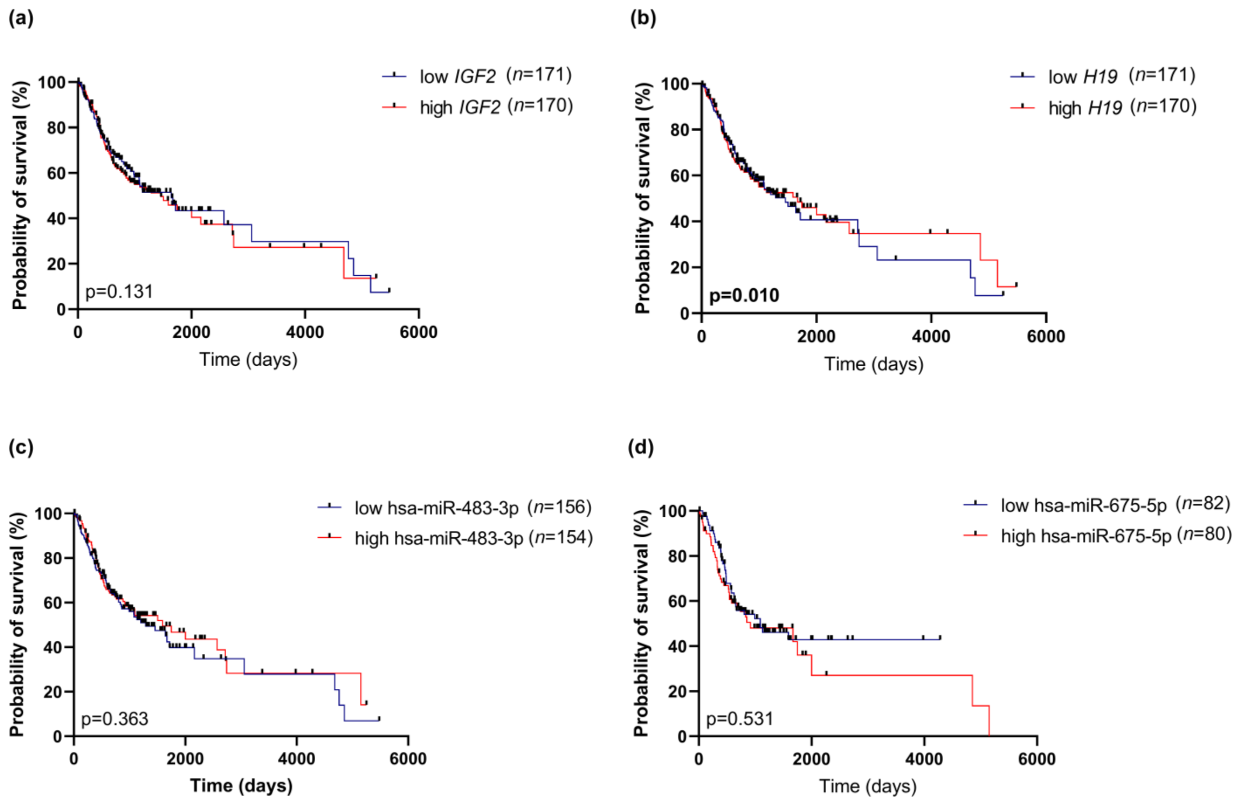

Figure S3. Kaplan–Meier curves of overall survival in oral cancer patients depending on *IGF2* (a), *H19* (b), hsa-miR-483-3p (c), and hsa-miR-675-5p (d) expression levels. All data were retrieved from the TCGA-HNSC dataset. Low and high levels of relative expression refer to values above or below the median.

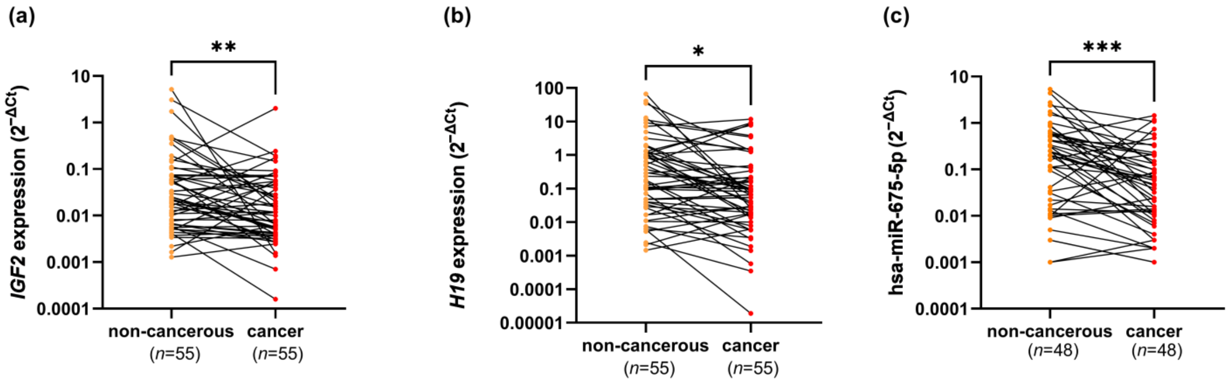

Figure S4. Expression profile of *IGF2* (a), *H19* (b) and hsa-miR-675-5p (c) in oral cancer and adjacent non-cancerous tissue – paired samples plot. The y-axis is on log10 scale. Statistical significance is shown with symbols: *p<0.05, **p<0.01, ***p<0.001

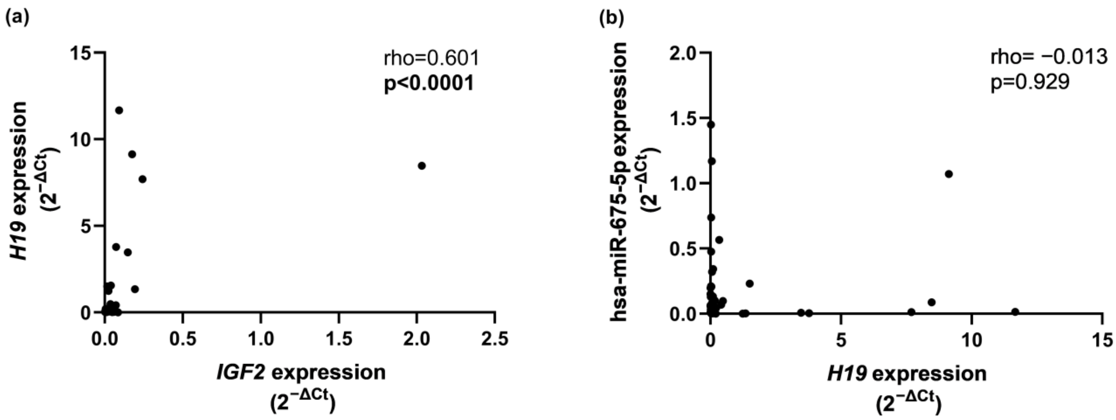

Figure S5. Correlation analysis between expression levels of *IGF2* and *H19* (a), and *H19* and its hosted hsa-miR-675-5p (b) in oral cancer clinical samples from our study group.
